# The Lichens’ Microbiota, Still a Mystery?

**DOI:** 10.3389/fmicb.2021.623839

**Published:** 2021-03-30

**Authors:** Maria Grimm, Martin Grube, Ulf Schiefelbein, Daniela Zühlke, Jörg Bernhardt, Katharina Riedel

**Affiliations:** ^1^Institute of Microbiology, University Greifswald, Greifswald, Germany; ^2^Institute of Plant Sciences, Karl-Franzens-University Graz, Graz, Austria; ^3^Botanical Garden, University of Rostock, Rostock, Germany

**Keywords:** lichens, symbiosis, microbiome, lichen-associated bacteria, *Lobaria pulmonaria*, omics

## Abstract

Lichens represent self-supporting symbioses, which occur in a wide range of terrestrial habitats and which contribute significantly to mineral cycling and energy flow at a global scale. Lichens usually grow much slower than higher plants. Nevertheless, lichens can contribute substantially to biomass production. This review focuses on the lichen symbiosis in general and especially on the model species *Lobaria pulmonaria* L. Hoffm., which is a large foliose lichen that occurs worldwide on tree trunks in undisturbed forests with long ecological continuity. In comparison to many other lichens, *L*. *pulmonaria* is less tolerant to desiccation and highly sensitive to air pollution. The name-giving mycobiont (belonging to the Ascomycota), provides a protective layer covering a layer of the green-algal photobiont (*Dictyochloropsis reticulata*) and interspersed cyanobacterial cell clusters (*Nostoc* spec.). Recently performed metaproteome analyses confirm the partition of functions in lichen partnerships. The ample functional diversity of the mycobiont contrasts the predominant function of the photobiont in production (and secretion) of energy-rich carbohydrates, and the cyanobiont’s contribution by nitrogen fixation. In addition, high throughput and state-of-the-art metagenomics and community fingerprinting, metatranscriptomics, and MS-based metaproteomics identify the bacterial community present on *L. pulmonaria* as a surprisingly abundant and structurally integrated element of the lichen symbiosis. Comparative metaproteome analyses of lichens from different sampling sites suggest the presence of a relatively stable core microbiome and a sampling site-specific portion of the microbiome. Moreover, these studies indicate how the microbiota may contribute to the symbiotic system, to improve its health, growth and fitness.

## Lichens: A Fascinating Symbiosis

### Introduction

**FIGURE 1:**
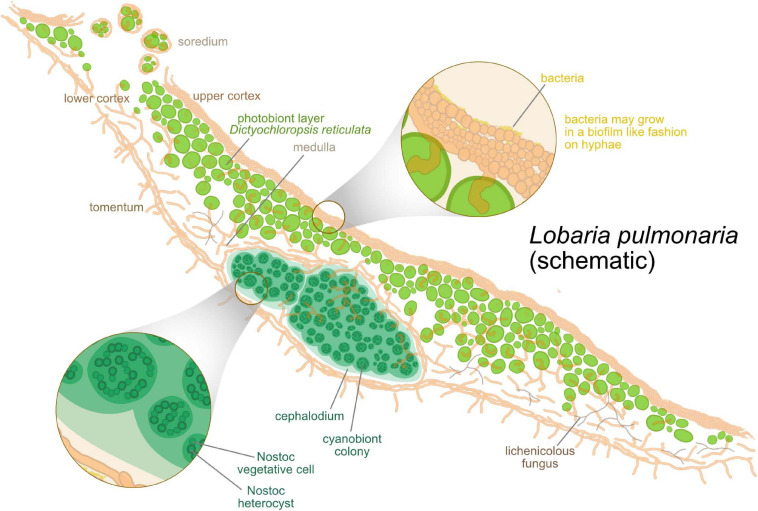
Schematic anatomical structure of lichens, with *Lobaria pulmonaria* as an example. Algae are hosted in a layered fungal scaffold structure delimited by an upper and a lower cortex. Green algae form a layer under the upper cortex. Below the algal layer, a hydrophobic medulla facilitates gas exchange within the thallus. As a tripartite lichen, *L. pulmonaria* hosts clusters of cyanobacteria inside the thallus in addition to the algae.

Evolution is replenished with examples of symbiotic life forms, which often include great examples of emergent metabolic solutions and joint morphologies. Being terrestrial symbioses and widespread in natural habitats, lichens are eye-catching examples that developed characteristic growth styles of their joined symbionts at least 415 million years ago (Honegger et al., 2013). According to the classic definition, lichens are a mutualistic relationship of one exhabitant heterotrophic mycobiont (i.e., a fungus, in most cases an ascomycete) and an autotrophic photobiont. Because the fungal partner determines the morphology of lichens, classification is integrated in the system of fungi. Accordingly, lichens may also be understood as fungi forming self-sustained life-forms with algae. The large majority of the approximately 20.000 described lichen species are formed with a green alga as photobiont, whereas only 10% of lichens represent symbiotic associations with cyanobacteria. Two to four percent of lichenized fungi may associate with both types of phototrophs (e.g., Figure 1; Hawksworth et al., 1995; Honegger, 1996). The latter are commonly shared in a single fungal individual, where green algae dominate and auxiliary cyanobacteria colonize specialized gall-like compartments, called cephalodia (Büdel and Scheidegger, 1996). A few species may even develop differently shaped morphologies (photosymbiodemes) with either of the photobiont types (Nash, 2008), which allow them to establish in different environmental situations (Honegger, 1996; Stenroos et al., 2003).

### Where Do Lichens Occur?

Lichens can grow on a broad range of substrates, yet, they are most common on bare rocks, on the bark of trees, or on compacted soil. Their habitats can be much more hostile than those favorable for higher plants (Kershaw, 1985; Walser, 2004). Despite being less productive, lichens can occur in rocky deserts or even at altitudes of up to 7400 m. Lichens can also form a major component of cool and humid habitats (e.g., Arctic tundra, boreal forest floors, lava fields, antarctic habitats, or rock surfaces in high altitudes or at the coast with tidal inundation). In certain ecosystems (e.g., tropical montane forests or temperate wet forests), epiphytic lichen biomass may exceed several hundred kilograms per hectare (Kershaw, 1985; Boucher and Stone, 1992; Coxson and Nadkarni, 1995). Yet, despite the outstanding tolerance of many species for desiccation, radiation, and extremes of temperatures in hostile environments, lichens are known as bioindicators of specific ecological situations. Apparently, lichens are very sensitive to ecological conditions when they are metabolically active, but they may tolerate extreme conditions when dry and metabolically inactive.

### Special Peculiarities of the Lichen Symbiosis

#### Morphology

Lichens develop a wide range of morphological shapes and growth styles including brushy, crust-like, or leaf-like forms, to name just the most important ones. Crustose thalli attach directly to the substrate with their entire lower surface, yet many species can also develop within the substrate (e.g., in sandstone, calcareous rocks, or bark). Surface-detached thalli often develop specific holdfast structures. In contrast to crustose forms, lichens with leaf-like growth styles are often devoid of degrading algal cell wall remnants in their upper fungal cortex layers. The apparent lack of expressing algal cell wall digestive functions in these species possibly gives their thalli structures higher hygroscopic flexibility and structural stability. For a long time, lichen systematists classified lichens exclusively according to their thallus morphologies or by details of reproductive structures, until molecular data revealed the phylogenetic relationships of those forms in better detail. Since then, molecular studies uncovered multiple cases of convergent evolution of similar growth styles, and the repeated evolution of surface-detached forms from crustose ancestors (Grube and Hawksworth, 2007).

The proper development of the vegetative lichen thallus with specific algal strains is also generally necessary but not always sufficient for sexual reproduction by fruitbodies of the mycobiont partner and/or for production of asexual/mitotic structures for joint propagation of both symbionts.

Most of the lichen thalli are rather brittle when dry, but rather flexible when wet. Some of those containing cyanobacteria may then have a rather gelatinous consistency as well (Büdel and Scheidegger, 1996).

The formation of tight, yet swellable fungal layers to wrap algal colonies, is apparently one of the key evolutionary innovations enabling lichen thallus evolution. The processes leading to gluing fungal hyphae together by their outer cell walls might be related to those involved in the formation of fruit body structures in ascomycetes. The interaction of the fungi with appropriate photobionts is required for the production of the characteristic thallus morphologies, but also for production of specialized metabolites, which accumulate - often at substantial amounts - as crystallized lichen compounds in the intercellular spaces of the thalli.

#### What Holds the Lichen Together?

Hydrophilic, extracellular substances (EPS; Figure 1; Spribille et al., 2020) achieve the gluing together of short, branched and anastomosing fungal hyphae. In many lichens, the protecting conglutinated fungal layer not only contains mycobiont hyphae, but also bacteria and basidiomycete yeasts. Due to its well-developed EPS and multiorganismal composition, this layer can thus be considered as a complex biofilm or as an extracellular interaction matrix (EIM; Spribille, 2018; Tuovinen et al., 2019). The EIM not only functions as a sort of exoskeleton for stabilization but also as a receptacle for mineral nutrients, a development zone for secondary metabolites and as medium of intercell recognition and signaling. As far as is known, the EIM of lichens contains different polysaccharides, which typically consist of D-glucose, D-galactose and D-mannose. The different polysaccharides, such as β- and α-glucans, α-mannans or heteroglycans, found in lichen mycobionts are discussed in several reviews (e.g., Olafsdottir and Ingólfsdottir, 2001; Spribille et al., 2020).

The Basidiomycete genus *Tremella* produces glucuronoxylomannans as exopolysaccharide capsule material (GXM; Gorin and Barreto-Bergter, 1983; De Baets and Vandamme, 2001; Martinez and Casadevall, 2015). No GXM is currently known from lichens, but *Tremella* yeasts are known from several lichens such as *Letharia vulpina* (Tuovinen et al., 2019). Therefore, Spribille et al. (2020) assume that GXM occurs also in lichens. This would agree with observations of Grube and De Los Rios (2001) who observed acidic polysaccharides on the outer cell walls of the lichen-associated *Tremella* relative *Biatoropsis usnearum*. Beside polysaccharides found in the EIM and secreted by fungal partners, additional ones are isolated from lichen algal photobionts, like amylose, β-xylan (Cordeiro et al., 2003), β-(1→5)-galactofuranan (Cordeiro et al., 2005, 2008), rhamnogalactofuranan (Cordeiro et al., 2013), mannogalactan (Cordeiro et al., 2010), cellulose or 4-linked mannan (Carbonero et al., 2005; Centeno et al., 2016). Additionally, some cyanobacterial EPS are known, such as β-(1→4)-xylan and a complex polysaccharide formed of β-linked L-arabinose and D-xylose (Ruthes et al., 2010).

The role of lichen-associated bacteria in EIMs will be discussed in sections “Uptake and supply of nutrients, essential compounds and trace elements” and “Microbiome Acquisition and Shaping.”

#### Poikilohydry: Life With Fluctuating Water Supply

Lichens are poikilohydric organisms meaning that they lack the ability to maintain and/or actively regulate their water content. Rather, their water status varies passively with the surrounding environmental conditions. In this regard, lichens differ from higher plants, where stomata and the cuticula help to manage the water balance. Therefore, lichens do not have a clear-cut separation between exo- and endosphere. As poikilohydric organisms, many lichens evolved extraordinary tolerance to desiccation. This includes antioxidant and photoprotective mechanisms (Kranner et al., 2005, 2008), and strategies enabling the lichen to cope with mechanical stress under changing hydration conditions. In dry conditions, the fungal layer consists of a dense EPS matrix and compressed hyphae. During lichen hydrating, the extracellular matrix expands by multiple times of its desiccated volume (Spribille et al., 2020). A more or less coherent layer that can flexibly respond to shrinking and swelling of the algal cells under variable conditions of hydration represents a main principle of the vegetative mycelium. The adaptation of some lichens to habitats with different water regimes also includes the choice of the photobiont. Green algae in lichens can activate their photosynthesis in a humid/moist environment whereas cyanobacteria in lichens usually require liquid water (Richardson, 2002). This explains why cyanolichens are more commonly found under conditions characterized by run-off water. Generally, the light and desiccation tolerance is facilitated by symbiotic interaction, thus ameliorating the stress faced by separated partners (Walser, 2004; Kranner et al., 2005; Jüriado and Liira, 2009; see also section “What **H**olds the **L**ichen **T**ogether?”).

#### Reproduction

Sexual reproduction of the algal partner is generally suppressed, but the fungal partner sexually reproduces commonly by production of meiospores in fruit-bodies. Fruit-bodies are usually perennial and can produce spores for many years under suitable circumstances, unlike those of many non-lichenized ascomycetes. Asexual, mitotic reproduction of the fungal partner is also known (e.g., thallospores, conidia). However, the fungal spores need to re-establish the symbiosis with a specific photobiont species, and despite the partners having the potential to grow autonomously (as they can be cultivated separately and are found by environmental sequencing), only the encounter of suitable photobiont initiates the ontogenetic processes to produce the characteristic thallus morphology. Therefore, lichens evolved also very diverse forms of asexual symbiotic propagules apart from the dispersal of fungal propagules alone (Figure 1). These structures range from dust-like or grainy microscopic particles containing few algal-encaging fungal hyphae (soredia) to more complex, stratified structures of different shapes and sizes (blastidia, isidia, schizidia, phyllidia, etc.) or small thallus fragments (folioles, squamules, etc.) that easily detach from the parental lichen thallus (Bowler and Rundel, 1975; Scheidegger, 1995).

#### Lichen Tolerance to Toxic Compounds

A remarkable feature of lichens (and many other macroscopic fungi) is that they are valuable and sensitive bioindicators of environmental and industrial pollution. Most lichens are sensitive to sulfur dioxide, which disrupts photosynthesis. Some lichens, however, have the ability to reduce the uptake of acidic precipitation containing sulfur dioxide. Hauck et al. (2008) pointed out that surface hydrophobicity is the main factor controlling the tolerance of these lichens for sulfur dioxide. Hydrophobicity could also explain that sulfur dioxide tolerant lichen species show a general toxin tolerance. As lichens also take up and tolerate metals in the thallus, they are used for environmental monitoring of industrial pollution (Garty, 2001). The production of metal oxalates (e.g., hydrated copper oxalate, manganese oxalate or lead oxalate) is a common adaptive biological mechanism for metal detoxification in lichens (reviewed by Purvis, 2014). Superficial oxalates on the thallus surface may help deflect the amount of light reaching the lichen photobiont and therefore help lichens to survive in extreme environments. An additional detoxification response to heavy metals is found in *Lecanora polytropa*. Phytochelatin (cysteine-rich peptides derived from glutathione) is produced to chelate these metals intracellularly (reviewed by Purvis, 2014).

### Symbiotic Partnership

#### Mycobiont: Home Sweet Home

The lichen-forming fungus develops the characteristic morphology of the lichen. It exposes the photobiont to controlled levels of sunlight during physiologically active stages, because a too strong light intensity can damage the photobiont (Figure 1; Larson, 1987).

A highly hydrophobic cell wall surface layer of mycobiont origin can be formed at the contact of the growing hyphae with a photobiont cell. This layer spreads over the wall surface of the entire algal cell, which seals the apoplastic space and channels the metabolic flux among symbiotic partners. Proteins, lipids and fungal derived phenolic secondary compounds form this hydrophobic coat. These secondary metabolites crystallize on and within the cell wall surface layer (Honegger, 1984, 1991; Scherrer et al., 2000).

The mycobiont is able to shift the position of the algal cells over short distances to secure adequate illumination and most efficient photosynthesis (Honegger, 1987, 1990). The hydration state of the cortical layer influences the light absorption and transmission. Additionally, the thickness of the cortical layer or the presence of insoluble mineral complexes, such as oxalates of calcium, copper, magnesium or manganese, and fungal-derived crystalline secondary compounds may affect the amount and spectral composition of incoming light (Honegger, 1991). Furthermore, the cortical layer has to facilitate the gas exchange of its algal photobiont (Honegger, 1990), which benefits from the position of the photobiont cells at the periphery of the gas-filled thalline interior underneath the fungal cortical layer. The cortex allows CO_2_ diffusion when the thalline water content is moderate, while water-supersaturated conditions may limit net photosynthesis rates significantly. As an adaptation to adequate level of CO_2_ absorption, a distinct proportion of the thalline volume [e.g., 30-50% in *Parmelia sulcata* (Fiechter, 1990)] acts as gas-filled intercellular space in the medullary and algal layers where mycobiont-derived respiratory CO_2_ can be stored (Honegger, 1991).

High light intensity can damage the photobiont (e.g., Demmig-Adams et al., 1990; Gauslaa and Solhaug, 1996, 1999, 2000). Depending on the long-term level of solar radiation, some lichenized fungi also produce melanic compounds in the outer layer of the upper cortex as a sunscreen. The melanic compounds reduce UV-B and UV-A wavelengths, but also visible wavelengths, reaching the photobiont layer. The melanisation leads to a browning of the cortex. This browning of the cortex is a physiologically active process, which occurs only in hydrated lichen thalli (Gauslaa and Solhaug, 2000, 2001; Solhaug et al., 2003; Antoine and McCune, 2004; Gauslaa and McEvoy, 2005; McEvoy et al., 2007; Nybakken et al., 2007; Matee et al., 2016; Mafole et al., 2017). As a side effect, melanins increase the absorbance of solar energy for the whole thallus resulting in a temperature increase of up to 3°C, which could suggest quicker water loss than in other species, which instead use crystallized secondary metabolites (e.g., atranorin) as light shields. Secondary metabolites appear as effective as melanin in reducing the transmission of photosynthetic active radiation, but reflect rather than absorb this radiation. Thus, these compounds have less effect on the heat balance of lichens (McEvoy et al., 2007; Solhaug et al., 2010; Mafole et al., 2017). Beside the production of melanic compounds or secondary metabolites, curling of the thalli during drying represents an additional strategy against serious photo-damaging thalli of *L. pulmonaria* during high light exposure (Barták et al., 2006).

#### Photobiont: The Breadwinner

The photobiont produces and secretes mobile energy-rich carbohydrates, which are provided to the fungal partner as sugar alcohols, such as polyols including ribitol, erythritol or sorbitol (Richardson et al., 1968; Hill, 1972; Brown and Hellebust, 1978; Smith and Douglas, 1987; Eisenreich et al., 2011; Eymann et al., 2017). The photobiont’s cell walls are permeable to carbohydrates in the lichenized state. Therefore, ribitol reaches the lichen fungus via diffusion (Richardson et al., 1968; Hill, 1976). The photosynthate release is probably stimulated by the synthesis and metabolism of taurine (Wang and Douglas, 1997). Taurine catabolism dioxygenase, which is an indicator for sulfonate utilization and metabolism of taurine, is identified in the proteome of the mycobiont of *L. pulmonaria* (Eymann et al., 2017). For storage, the mycobiont partly converts the carbohydrates provided by the photobiont into mannitol via the pentose phosphate pathway (Smith, 1980; Lines et al., 1989; Eisenreich et al., 2011). Polyols cannot be regarded as safe storage products because they can be washed out of the thallus during rewetting after drought stress (MacFarlane and Kershaw, 1985; Dudley and Lechowicz, 1987). Polyols more likely protect enzyme systems during stress events and act as osmoregulators during the wetting and drying cycles. Additionally, they can be substituted for water and stabilize proteins and membranes under dry conditions (Bewley and Krochko, 1982; Farrar, 1988; Jennings and Lysek, 1996). This raises the interesting hypothesis that the green algae originally synthesize the sugar alcohols only as compatible solutes mainly just to keep water in the cells and to protect their proteins under dry stress conditions. Rapid rewetting, e.g., by rain, could force the green algae to export polyols very rapidly to prevent uncontrolled water influx and cell damage. Involuntarily released sugar alcohols could thus possibly played a role in early stages of lichen evolution.

When the lichen thallus is drying the water from the apoplastic space between the cell walls of the symbiotic partners is lost at first. Afterward, cellular water is partially lost, leading to drastic but reversible cell shrinkage. The mycobiont and photobiont cells are able to tolerate dramatic fluctuations in cellular water contents between saturation (> 150% water content in relation to dry weight) and desiccation (< 20% water content in relation to dry weight). Soluble compounds are passively released into the apoplastic space due to reversible leaching from the cell during extreme drought. Lichen photobionts reversibly inactivate their photosystem II during desiccation. Water flows passively back and forth within the apoplastic space during the regularly occurring de- and rehydration of the thallus. Thereby, not only dissolved mineral nutrients, but also other passively and actively released metabolites from the symbiotic partners are translocated (MacFarlane and Kershaw, 1985; Brown et al., 1987; Dudley and Lechowicz, 1987; Honegger, 1991, 1997; Scheidegger, 1994; Heber et al., 2010).

Eymann et al. (2017) assign beta-catenin to the proteome of the algal photobiont of *L. pulmonaria*. It is important for the signaling during cell-cell adhesion of unicellular organisms (Abedin and King, 2010), in the case of lichens between cells of the mycobiont and photobiont or among the photobiont cells and allows a rapid exchange of signals and substrates.

#### Cyanobiont: Grabbing Nitrogen

In tripartite lichens, the cyanobiont is predominantly responsible for nitrogen fixation (Millbank and Kershaw, 1970). This is supported by the identification of glutamate synthase and molybdenum nitrogenase in the proteome of the cyanobiont of *L. pulmonaria* (Eymann et al., 2017). In other lichens, such as *Peltigera aphthosa*, alternative nitrogenases, which use vanadium or iron instead of molybdenum at the active site, might also play an important role in biological nitrogen fixation lichens (Darnajoux et al., 2017). Because oxygen is inhibiting denitrogenase, the fungal partner creates microaerobic conditions and accumulates the cyanobiont cells in gall-like structures, also known as cephalodia (Jordan, 1970; Hawksworth and Hill, 1984; Jahns, 1988; Honegger, 1993; Büdel and Scheidegger, 1996; Cornejo and Scheidegger, 2013). Nitrogen fixation of the cyanobionts of lichens is substantial for nitrogen cycling in nutrient-poor environments where nitrogen leaks from growing and degrading lichens (Nash, 1996). In tripartite lichens, the cyanobacterial heterocyst frequency is increased. In bipartite lichens, the heterocyst frequency varies between 2% and 8%, whereas in tripartite lichens, it varies between 10% and 55%. Nevertheless, the relative number of cyanobacteria is kept lower than that of the algal photobiont. Thus, the lichen fitness increases through specialization of the cyanobiont on nitrogen fixation (Hyvärinen et al., 2002).

Although labor partition favors the role of nitrogen fixation in cephalodiate cyanobionts, the cyanobiont is also partly responsible for carbon fixation and thus, involved in photosynthesis. Cyanobacteria as well as red algae have a light-harvesting complex, which is different to that of green algae, the phycobilisome. This complex is built up of phycobilins, like phycoerythrin and phycocyanin. The phycobilisome absorbs light between 450 nm and 650 nm and sometimes beyond 700 nm. Chlorophyll a and b absorb light between 400 and 480 (blue light) and between 550 and 700 (yellow to red light). Therefore, green light is rarely absorbed by the green algal photobiont, whereby tripartite lichens and chlorolichens appear greenish (Kadereit et al., 2014; Adir et al., 2019). The existence of phycobilisome proteins is reported in the proteome of the cyanobiont of *L. pulmonaria* (Grube and Berg, 2009; Eymann et al., 2017).

#### Mycobiome: The Recycler, Probably?

The longevity and slow growth of lichen structures may also foster the colonization of lichens by more or less specific additional microorganisms. Lichenicolous (= lichen colonizing) fungi (Figure 1) were actually recognized even before the symbiotic nature of lichens was discovered in the second half of the 19th century. About 1800 lichenicolous fungi are known today and most of these are characterized by their morphological structures^1^ (Lawrey and Diederich, 2003; Diederich et al., 2018). Each lichen can harbor a wide range of additional fungal components, which constitute an associated mycobiome (Fernandéz-Mendoza et al., 2017; Muggia and Grube, 2018). The majority of the lichenicolous fungi only cause local infections in the host thallus or are more or less commensalic. The commensalic behavior usually correlates with a preference for the algal photobiont of the host lichen. Recent evidence suggests that commensalism also applies to yeasts resident in fungal layers of the lichen. Spribille et al. (2016) assumed that commensalic basidiomycete yeasts residing in the fungal upper cortex maintain close associations with specific lichen species over large spatial distances. This view was questioned by recent evidence of low host specificity of yeast stages compared to the known mycelial lichenicolous fungi. According to Mark et al. (2020), various epiphytic lichen species growing on the same tree trunk consistently harbor specific *Trebouxia* lineages as photobionts, while genotypes of the cystobasidiomycetous yeasts were irregularly distributed among the species.

Lichenicolous fungi contact the cells of their hosts using specific structures. De Los Rios and Grube (2000) described infectious hyphal structures, including simple or complex haustoria with projections into the host hyphae. Other species were found to use haustoria to penetrate the algal photobiont of their hosts (*Zwackhiomyces*; Grube and Hafellner, 1990). Furthermore, some lichen-associated fungi have necrotic to saprobic life styles, preferring decaying parts of lichens (Aptroot and Alstrup, 1999). Other lichenicolous fungi can cause hypertrophic deformations (galls) in lichen thalli, which usually contain hyphae of both fungal species. These galls also provide a microhabitat for additional fungi, including the yeast genus *Cyphobasidium* (Millanes et al., 2016).

Only a small fraction of lichenicolous species actually cause dramatic damage to their hosts and may rapidly erase lichen coverage of substrates (including the facultative lichen pathogen *Athelia arachnoidea*).

Lichenicolous fungi, which use structures of the host to establish their own lichenized thalli are called lichenicolous lichens (Poelt, 1990). Their parasitic thalli develop on the surface or as internal structures of the host (Grube, 2018), and they often also take up the algae of the hosts. Because the lichenicolous lichens can also be infected by lichenicolous fungi, lichens also provide interesting examples of hyperparasitism. For example, the lichenicolous lichen *Rhizocarpon diploschistidina* parasitizes the lichen *Diploschistes muscorum*, which usually grows as a juvenile parasite of *Cladonia* species (Lumbsch et al., 2011).

#### Microbiome: The New Teammate

Lichen-associated bacteria were initially isolated in the first half of the 20th century (Cengia Sambo, 1926; Henckel and Yuzhakova, 1936; Honegger, 1990). These characterizations already indicated a possible role in nitrogen fixation for some of these lichen-associated bacteria. Evidence for the presence of bacteria (other than cyanobacteria) in lichens is provided by a series of studies that are based on morphological evidence using cultivable isolates of bacteria in lichens (e.g., Panosyan and Nikogosyan, 1966; Henkel and Plotnikova, 1973). In the early 1980s, Lenova and Blum (1983) estimated that millions of bacterial cells per gram could colonize a lichen thallus. More than 20 years later, diversity and specificity was studied in more detail by culture-independent sequencing approaches, and later by more sophisticated omics technologies, which demonstrated that lichens are furnished with a complex bacterial microbiome (Figure 1) (e.g., González et al., 2005; Cardinale et al., 2006; Liba et al., 2006; Grube et al., 2009; Selbmann et al., 2010; Bates et al., 2011; Hodkinson et al., 2012; Aschenbrenner et al., 2014). The lichen microbiome is identified as a surprisingly abundant and structurally integrated element of the classical lichen symbiosis (e.g., Gauslaa and Solhaug, 1996, 1999; Aschenbrenner et al., 2016; Leiva et al., 2021). These studies reveal that lichen-associated bacterial communities are not merely a simple extension of the prokaryotic community of the lichen-surrounding environment. The lichen microbiome consists of two parts. The first part is a core microbial community. Therefore, the microbiome is stable to a certain extent. The second part of the microbiome is a specific microbial community, even in close spatial proximity or when lichens are reshaped by parasitic invasion of one lichen into the other (Wedin et al., 2016). Thus, the lichen species is probably the best predictor of its microbiome composition (Bates et al., 2011). These findings led to reconsideration of the lichen symbiosis including the microbiome as an additional component (Hawksworth and Grube, 2020).

### Lichen-Associated Viruses

Reports of lichen-associated viruses are found in recent studies. In several lichens, single and double-stranded RNA viruses, similar to those of plants were found (Petrzik et al., 2014, 2016). Yet, lichen viruses are not only of apparent algal origin. Petrzik et al. (2019) detected novel dsRNA viruses in the lichens *Chrysothrix chlorina* (Chrysothrix chrysovirus 1; CcCV1) and *Lepraria incana* (Lepraria chrysovirus 1; LiCV1) and classified them to the genus *Alphachrysovirus*, with a relationship to chrysoviridae from filamentous ascomycetous fungi. However, the authors showed that CcCV1 was not found in the lichen mycobiont but in the accompanying endolichenic fungus *Penicillium citreosulfuratum*. Using dsRNA-seq technology, Urayama et al. (2020) characterized the total dsRNA viral community of a lichen species (species name not indicated, but apparently belonging to the *Cladonia pyxidata* complex according to their Figure 1), and revealed that partitiviruses were dominant and active. Sequences found in this study were classified into two genera, which include both plant- and fungi-infecting partitiviruses. Apparently, each of the lichen partners may harbor several virus species independently and simultaneously because CaMV and the capsid protein gene of ApMV were detected in the photobiont of *Xanthoria parietina* and both, the plant cytorhabdovirus and the ApMV, were detected in *U. chaetophora* (Petrzik et al., 2014, 2015). Also, proteins assigned to rhabdoviruses and betaflexiviruses were found in the metaproteome of *L. pulmonaria* (Eymann et al., 2017), and Grube et al. (2015) found bacteriophage sequences in the metagenome of the same lichen. Presence of bacteriophages in lichens is confirmed by occurrences of bacteriophage proteins assigned to the families Myoviridae and Siphoviridae which infect Bacteria and Archaea (Eymann et al., 2017).

## The Prokaryotic Microbiome of Lichens

### Bacteria Living With Lichens

More than 800 types of bacteria can contribute to the bacterial microbiome of a single lichen individual. In many so far studied lichens, Alphaproteobacteria form the largest and metabolically most active bacterial class (Cardinale et al., 2006, 2008; Grube and Berg, 2009; Grube et al., 2009, 2015; Bates et al., 2011; Schneider et al., 2011). Within the Alphaproteobacteria, the Rhizobiales make up the majority. In chlorolichens, Rhodospirillales are often co-dominant contrary to cyanolichens where Sphingomonadales normally co-dominate. However, Alphaproteobacteria are not always dominant. The rock-inhabiting lichen *Ophioparma* is dominated by Acidobacteria (Hodkinson et al., 2012), and marine lichens (e.g., *Lichina pygmaea*) differ by a dominance of Bacteroidetes beside Chloroflexi and Thermi (West et al., 2018). Due to their preference to form communities with fungi and in addition with Alphaproteobacteria on plant surfaces, it is not surprising to find also *Paenibacillus* and *Burkholderia* phylotypes within the microbial community of terrestrial lichens (Berg and Hallmann, 2006; Cardinale et al., 2006; Grube and Berg, 2009; Partida-Martinez et al., 2007). Other well-known bacterial lineages can be found in lichens such as Firmicutes, Bacteroidetes, Verrumicrobia, Acidobacteraceae, Acetobacteraceae, Brucellaceae or Chloroflexi (González et al., 2005; Grube et al., 2009; Bates et al., 2011; Schneider et al., 2011; Hodkinson et al., 2012; Garg et al., 2016).

**FIGURE 2:**
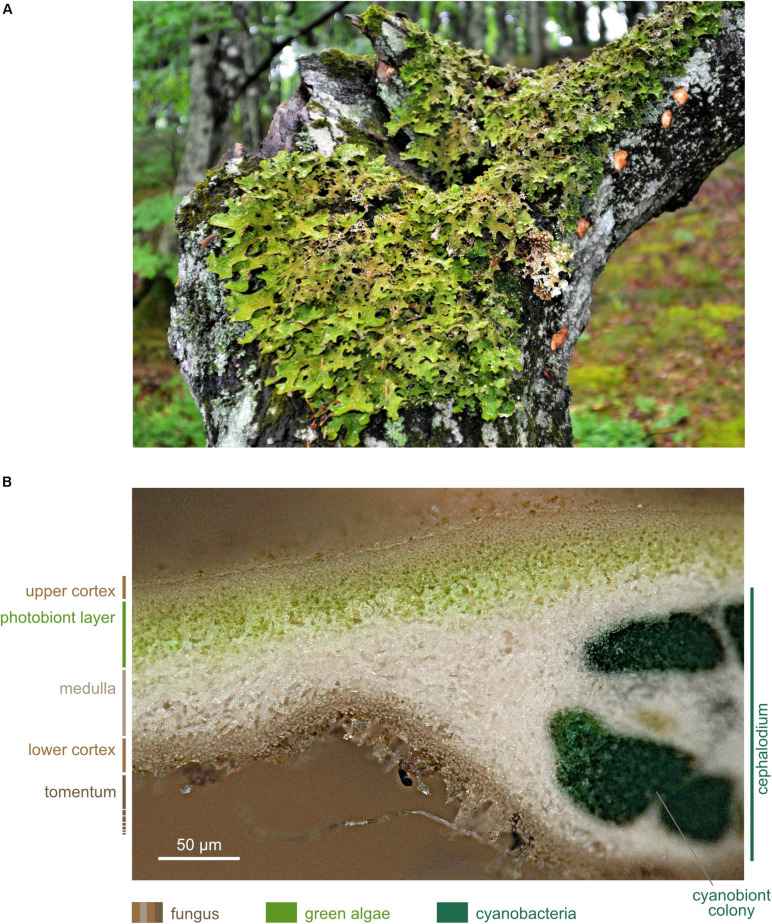
*Lobaria pulmonaria*. **(A)** Specimen growing on *Fagus sylvatica* (photographed in May 2016 near Viborg, Denmark); **(B)** Microscopic cross section. The upper cortex forms the habitat of the microbiome. Image produced with dry, sectioned material using a Keyence Digital VHX-5000 microscope with automated image stacking.

So far, most microbiome studies focused on lichens belonging to Lecanoromycetes. One of them is the lung lichen *L. pulmonaria* L. Hoffm. (Figure 2) and is currently under intense investigation. One third of the overall bacteria in *L. pulmonaria* belong to the Rhizobiales (in particular to the families Methylobacteriaceae, Bradyrhizobiaceae, and Rhizobiaceae), which are well known partners in plant-microbe interactions (Erlacher et al., 2015). Actinobacteria, Betaproteobacteria, Firmicutes or Deinococcus (Cardinale et al., 2006; Hodkinson and Lutzoni, 2009) and Archaea are complementing the *L. pulmonaria* microbiome (Schneider et al., 2011; Eymann et al., 2017). *L. pulmonaria* is an interesting model to investigate genotypic and phenotypic traits that enable the lichen to adapt to changing climate conditions. In the following paragraphs, we will present a more detailed look at *L. pulmonaria* and its microbiome together with the microbiome of other lichen species. Moreover, we will introduce important techniques for studying composition and function of lichen microbiomes.

### The Model Lichen *L. pulmonaria*

*Lobaria pulmonaria* is a large foliose lichen (Figure 2A). Besides a myco- and a photobiont, *L. pulmonaria* contains a cyanobiont, a cyanobacterium belonging to the genus *Nostoc*. The green algal photobiont *Dictyochloropsis reticulata* dominates 90% of the subcortical layer of the thallus (Honegger, 1991; Myllys et al., 2007; Figure 2B).

**FIGURE 3:**
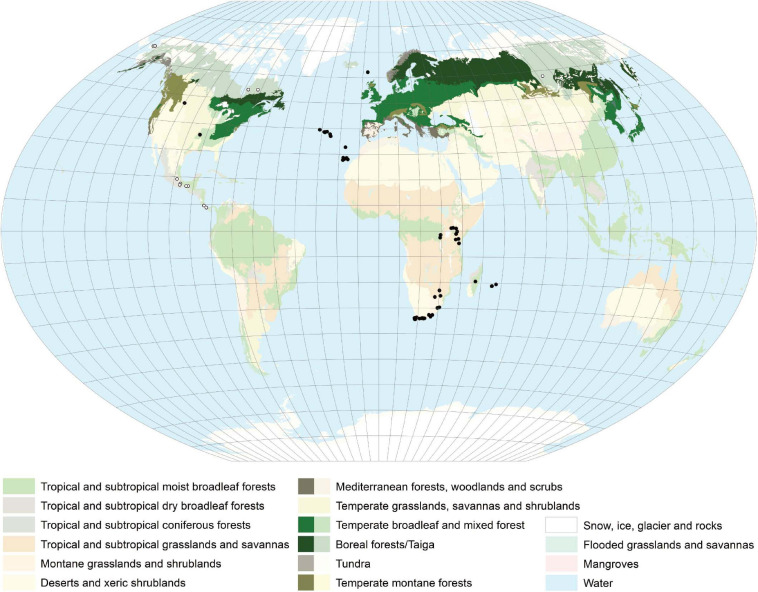
Distribution map of *L. pulmonaria* created with ArcGIS 10. Colors indicate the different vegetation zones. Strongly colored areas – general distribution regions. Solid dots – single occurrence spots. Open dots – occurrence not clarified. *L. pulmonaria* has a worldwide distribution mainly occurring in the Northern Hemisphere.

*Lobaria pulmonaria* is distributed over an area in the Northern Hemisphere from the boreal to the meridional zone (Figure 3, GBIF.org 2019). In Eurasia as well as in Northern America, it has a Western and an Eastern subarea, excluding regions influenced by a strong and dry continental climate with precipitation less than (450)-500 mm. Furthermore, *L. pulmonaria* occurs in the montane belt of East Africa, on Madagascar, the Mascarene archipelago and in South Africa (Figure 3). Occurrences in Central America still merit further investigation and need to be validated. The geographic range reflects the ability of *L. pulmonaria* to inhabit regions with different overall conditions. The mean annual temperature at the Northernmost locations in Scandinavia is about 1°C and the mean annual precipitation about 500 mm, on the Azores archipelago about 18°C and 1.500 mm, respectively, and in Siberia at the Baikal lake −0,9°C and about 500 mm, respectively (Worldclim 2.0). Consequently, *L. pulmonaria* occurs in a wide spectrum of habitats mostly on tree trunks, e.g., *Fagus sylvatica*, *Quercus robur* or *Acer platanoides* (Hakulinen, 1964; Wirth, 1968; Hallingbäck and Martinsson, 1987; Rose, 1992; Wolseley and James, 2000; Kalwij et al., 2005; Jüriado and Liira, 2009; Smith et al., 2009; Wirth et al., 2013), but it can also colonize dwarf scrubs (e.g., *Calluna*), mossy rocks and soil (Ingólfsdóttir et al., 1998; Scheidegger and Goward, 2002; Grube et al., 2015). The current distribution of *L. pulmonaria* is the result of several factors. Air pollution by sulfur dioxide (e.g., Hawksworth and Rose, 1970; Hawksworth et al., 1973; Hallingback and Olsson, 1987; Farmer et al., 1991), and later, by nitrogen containing air pollutants (e.g., Hauck and Wirth, 2010) led to extinction or a reduced frequency in many regions of the original distribution area. The forestry practices strongly influenced *L. pulmonaria* in different respects for a long time. Clear cutting and afforestation with trees not suitable to the location (e.g., spruce, pine) led directly to the loss of habitats, other forestry measures to negative changes of the habitat conditions (e.g., Edman et al., 2008; Jüriado et al., 2011; Wirth et al., 2011; Schiefelbein and Thell, 2018). In conclusion, *L. pulmonaria* occurs mostly in undisturbed natural forests, with long ecological continuity due to a stable environment in terms of light, moisture and temperature, which are little influenced by air pollution, agriculture and forestry practices.

Under suitable conditions, *L. pulmonaria* may have a long life (acc. to estimates up to 200 years) (Scheidegger et al., 1998) and is described as a ‘patchtracking’ organism (Snäll et al., 2003), colonizing tree boles, where it may persist until the tree dies or microclimatic conditions change. During the life cycle of *L. pulmonaria*, estimated to an average of 35 years, the dispersal via vegetative diaspores (soredia, isidioid soredia) or thallus fragments predominates (Scheidegger et al., 1998; Scheidegger and Goward, 2002). Molecular and empirical studies show that *L. pulmonaria* can efficiently propagate by their vegetative propagules up to 75 m (Walser et al., 2001; Öckinger et al., 2005; Werth et al., 2006), but the majority of *L. pulmonaria* vegetative propagules are detected at a very short spatial scale, i.e., < 40 m (Werth et al., 2006). Under proper conditions (which usually agrees with larger thallus sizes), fruitbodies and ascospores develop (Scheidegger and Goward, 2002; Martínez et al., 2012; Wirth et al., 2013) and facilitate the long-distance dispersal of the mycobiont (Werth et al., 2006). With up to 5 mm annual tip growth, *L. pulmonaria* is one of the fastest growing lichens in Europe (Phillips, 1969). Microsatellite analysis by Werth and Scheidegger (2012) reveal dispersal via symbiotic propagules as the key factor shaping the genetic structure of this species. The highest growth rates and shortest generation times, with reproductive maturity are achieved within around five to ten years, as Eaton and Ellis (2014) observe in an oceanic hazelwood in Western Scotland. Thus, in oceanic environments, e.g., along the west coast of Scotland (Seaward, 1998; Eaton and Ellis, 2014), *L. pulmonaria* is very common. The difference in abundance between oceanic and more continental environments possibly reflects an interaction between the macroclimate and an organisms’ microhabitat specificity (Lisewski and Ellis, 2010), such that a species may become increasingly restricted to a limited suite of buffered microclimatic niches under sub-optimal macroclimatic conditions (Doering and Coxson, 2010), and therefore rarer.

Because of its specific features and threats, *L. pulmonaria* is extinct or at risk in many regions of its distribution area (e.g., Türk and Hafellner, 1999; Scheidegger and Clerc, 2002; Bardunov and Novikov, 2008; Randlane et al., 2008; Farkas and Lõkös, 2009; Aptroot et al., 2012; Wirth et al., 2011).

This lichen species is used as a model lichen for studies on the lichen microbiome mainly due to its relatively fast growth and its ecological significance.

### Molecular Tools to Investigate Lichen-Associated Microorganisms

#### The Rise of ‘Omics’ Technologies

Lichen-associated bacteria were initially identified and investigated describing bacteria by their phenotypical and physiological characterizations (Cengia Sambo, 1926; Iskina, 1938; Panosyan and Nikogosyan, 1966; Henkel and Plotnikova, 1973; Lenova and Blum, 1983). Later, molecular techniques using bacterial isolates were conducted (González et al., 2005; Cardinale et al., 2006; Liba et al., 2006; Selbmann et al., 2010). However, culture-dependent methods generally capture only a minor fraction of the bacterial diversity of environmental samples (Amann et al., 1995). Therefore, new techniques were elaborated and the first culture-independent investigations, such as fingerprinting methods (Cardinale et al., 2006, 2012a; Grube et al., 2009; Bjelland et al., 2011; Mushegian et al., 2011) or molecular cloning approaches (Hodkinson and Lutzoni, 2009), were started to generate microbial community profiles. A further advantage of these methods is the possibility to analyze many samples in parallel and to compare their profiles consistently. Using the pyrosequencing method, several DNA-based studies described lichen-associated bacterial communities (Bates et al., 2011; Grube et al., 2012; Hodkinson et al., 2012; Aschenbrenner et al., 2014).

**FIGURE 4:**
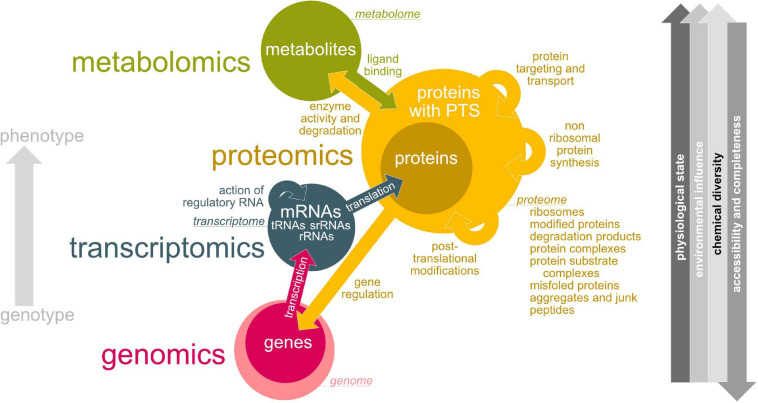
Overview about ‘-omics’ technologies used for lichen microbiome research. Green – metabolomics, yellow – proteomics, blue – transcriptomics, pink – genomics. Colored arrows indicate interactions between metabolome, proteome, transcriptome and genome and how they are affecting each other. Circle sizes illustrate estimated complexity. Gray arrow direction (right hand side) implies an increase.

By improvement of the sequencing methods and bioinformatic tools, research focus shifted to a more detailed and holistic view on lichen microbial communities. In this respect, the lichen microbiome can be studied using a diverse spectrum of ‘omics’ technologies (Figure 4). Despite metagenomic analyses (e.g., Grube et al., 2015; Cernava et al., 2015; Aschenbrenner et al., 2017; Cernava et al., 2018; Graham et al., 2018), many studies applied metatranscriptomic (e.g., Cernava et al., 2017, 2019) and metaproteomic (e.g., Berg et al., 2011; Schneider et al., 2011; Grube et al., 2015; Cernava et al., 2017; Eymann et al., 2017; Ullah et al., 2020) approaches to study lichen symbioses.

#### (Meta)Genomics: Accessing Life’s Blueprint

The total genetic information (encoded by DNA) of an organism is defined as its genome and all genomes within a habitat form the habitat’s metagenome. Despite long-term evolutionary processes the environment has only minor influence on the DNA structure and information content (Figure 4). Due to DNA’s stability and its low chemical diversity, methods for extraction, preparation and sequencing became routinely applicable (Figure 4). Genomics reads, assembles, stores and interpretes the DNA-encoded information and is important to understand the organization, structure and function of genes of an organism.

Metagenomic analyses of lichen microbiomes (Cernava et al., 2015; Aschenbrenner et al., 2017; Cernava et al., 2018; Graham et al., 2018) identify (gene)sequences covering a wide range of potential bacterial functions with possible impact on the symbiotic system and their assignments to the taxa which are present. However, metagenomics does not provide information whether these functions are specifically expressed by any of these bacteria under the given environmental conditions. However, this knowledge is required to understand how the microbiome responds to changing conditions in the lichens natural habitats.

Metagenomic analyses are currently dominated by high throughput sequencing of relatively short reads, but they are shorter than most genes and thus, read-based methods cannot provide sufficient information about the genomic organization of genes. Huge amounts of previously uncharacterized genomic diversity can be unveiled by creating metagenome-assembled genomes (MAGs; Parks et al., 2017; Maguire et al., 2020). Thereby, contigs are assigned to clusters or so-called “bins” based on their similarities in relative abundance and sequence composition parameters such as frequencies of nucleotide tuples (di-, tri-nucleotides). The creation of MAGs including tools are reviewed by Sangwan et al. (2016). The MAG approach has been successfully applied to a wide range of environments, e.g., aquatic habits (Narasingarao et al., 2012). For the first time, Wicaksono et al. (2020) reconstructed 29 bacterial MAGs (contamination < 10%) from metagenomes of lichens (17 MAGs: *L. pulmonaria*, 7 MAGs: *C. furcata* and 5 MAGs: *P. polydactylon*). Most of the created MAGs were assigned to Proteobacteria. This study indicates that creating MAGs can be crucial for further exploration of the ecological role of bacterial symbionts in lichens. The comparison of metagenomic data with those derived from other ‘omics’ techniques reveal that only a fraction of bacteria is active in lichens at the same time (Grube et al., 2015). Furthermore, MAGs could help analyze the utilization potential of complex carbohydrates by bacteria (Hassa et al., 2018).

#### (Meta)Transcriptomics: Determining Active Genes

The transcriptome is the total RNA transcribed from the genome of an organism at a given time point. Thus, the composition of the transcriptome directly reflects the entirety of external environmental stimuli on gene expression level (Figure 4). Due to the lack of available complete (meta)genomes only RNASeq (the determination of short cDNA sequence reads) is applicable for metatranscriptome studies of more complex biological systems such as lichens. Nevertheless, the availability of reference genomes would dramatically support the differentiation of transcripts between host and microbial RNA (Aguiar-Pulido et al., 2016).

Environmental metatranscriptomics focuses on mRNA to estimate the expressed genes in a given community and thus, helps to identify active metabolic pathways functioning under specific environmental conditions.

Cernava et al. (2017) analyzed the functional diversification of the microbiome of *L. pulmonaria* using a meta-omics approach. By using metatranscriptomics in addition to metagenomics and -proteomics facilitate the detection of so far unknown bacterial species in the lichen symbiosis. Metatranscriptomics as well as metagenomics revealed various strategies of lichen-associated bacteria to survive under stress conditions. The data derived from metaproteomic analyses can validate and corroborate metagenomic or -proteomic results.

Recently, Cernava et al. (2019) studied the role of lichen-associated microorganisms (*L. pulmonaria*) in enduring dehydration and drought using a metaproteomics approach. They revealed that the microbiome is well-adapted to dehydration by stress protection and additionally changes of the metabolism. Furthermore, an interplay in holobiont functioning under drought stress is indicated by their results.

The high abundance of ribosomal RNA in mRNA preparations may reduce the RNASeq coverage and the instability of mRNA in general has to be considered appropriately in metatranscriptomics.

#### (Meta)Proteomics: Seeing the Blueprint at Work

Proteins are synthesized by translating the available and intact mRNAs (Figure 4). By this reason, only the complement of active genes (mRNAs) also occurs on protein level. According to varying sequence length, the protein composition from 20 amino acids recombines the amino acid’s chemical properties. After translation, proteins may undergo wanted but also arbitrary chemical modifications such as phosphorylations and many others, site-specific cleavage, aggregation with other proteins or prosthetic groups. Transport processes may remove proteins from the analyzed sample. Unstable proteins will be rapidly degraded while proteins with long half lifes will last from several days until years. This results in protein-to-protein concentration differences covering several orders of magnitude. All of this impacts single protein molecules but also the composition of the protein pool at all. This explains why the proteome - the entirety of all proteins of a biological system at a given time point at a defined developmental stage under defined environmental conditions - is one of the most complex and dynamic biological entities under investigation we may need to handle. Covering all proteins in one experimental setup for the analysis of (meta)proteomes is not possible. Especially less abundant proteins below detection threshold and proteins tending to form complexes, to precipitate, or to bind to surfaces of sample particles or lab equipment may escape analysis or make an enrichment or adaptations of sample preparation protocols necessary.

For liquid chromatography mass spectrometry (LCMS) based proteomics, extracted proteins are enzymatic cleaved for generating peptides which subsequently can be ionized and mass-analyzed (Karpievitch et al., 2010). With tandem MS (MSMS) devices sequencing of higher abundant peptides is performed. The determined masses, sequence tags and signal strengths of peptides are then assigned to protein sequences.

For correctly assigning peptide data to proteins, a suitable bioinformatic workflow including the selection of an appropriate database is of fundamental importance (Heyer et al., 2017). Such databases are made from a collection of protein sequences, which are expected to be found in the analyzed sample. Usually one considers protein sequences from taxonomic and metagenomic analyses of comparable samples (Kunath et al., 2019). Including sequenced mRNA in the database allows us to focus on the active part of the genes/community (Hassa et al., 2018; Kunath et al., 2019). If only sequences of model organisms or any other biased data were used for database construction the peptide masses of unknown taxa will produce incorrect sequence hits or no hits at all.

After assignment of measured mass signals and sequence tags to peptides and peptides to proteins, the total quantities of single proteins by summing up all peptide signals is possible. The final result per sample will have determined the quantitative distribution of proteins. Species specific and functional protein assignments may reveal, which constituents of an analyzed sample are specialized to fulfill specific functions (Eymann et al., 2017). Per taxon quantification of all assigned proteins may show, to which extent each taxon contributes to the whole system on protein level (Eymann et al., 2017).

Putting the data of a set of samples together may allow binary sample-to-sample comparisons or the extraction of protein expression profiles along spatial or other environmental gradients. Such kind of differential metaproteome analysis could help for understanding the responses of microbial communities as an entirety to changing conditions and can thus provide information about community resistance and resilience under environmental stress. Schiebenhoefer et al. (2019) discuss appropriate bioinformatic tools for metaproteomic analysis.

More recent developments which are based on differential detection of peptides with different stable isotope composition offer fascinating opportunities to elucidate metabolically active taxa and metabolite fluxes within microbial communities (for an outlook see Kleiner, 2019). For stable isotope probing (SIP) labeled substrates such as sugars and carbon compounds or amino acids containing ^13^C, ^15^N, ^18^O or ^36^S respectively are applied. Active cells take up the labeled substrates, which can be later detected within the proteins via MS. The rate of incorporation in defined taxa of the investigated sample community can be used for assessing their metabolic activity or their preferences for the applied substrate (Seifert et al., 2012; Von Bergen et al., 2013).

Stable isotope fingerprinting (SIF) is an approach similar in principle, but circumvents the artificial application of labeled substrates. Due to naturally occuring carbon isotope discriminative effects against ^13^C in C1 pathways (e.g., for assimilation of bicarbonate, CO_2_, or methane), metabolites and biomass originating from these pathways show an altered carbon isotope (^13^C/^12^C, commonly called δC) ratio that can be used to reveal chemical fluxes and dependencies within metaproteome samples (Jehmlich et al., 2016). The software Calis-p supporting the direct extraction of species-specific δCs from standard metaproteome datasets was presented and made available (Kleiner et al., 2018). To our knowledge, SIP as well as SIF were not applied to lichens until now.

#### Metabolomics: Analyzing Enzyme’s Action

Proteins interact with each other resulting in a complex metabolic system of an organism. Changes in the proteome lead to changes in the metabolome. Thus, the metabolome is highly dynamic and complex (Figure 4). Changes can develop in fractions of seconds making metabolome analysis and sample preparation a very challenging task. Metabolites can additionally act as cofactors, signaling molecules, substrates or stabilizing agents for proteins. Metabolomics determines a sample’s profile of metabolites at a defined time point under certain environmental conditions (Figure 4). Metabolomics may play a role to examine the available metabolites of the lichen and its microbiome.

Antibiotic effects of a number of lichen metabolites against gram-positive bacteria is known since long (reviewed, e.g., in Shrestha and Clair, 2013), and presumably, such compounds could regulate bacterial growth in lichens. A disadvantage of metabolomics in contrast to metaproteomics is the fact that it is challenging to assign metabolites to specific taxa, due to the lack of taxon-specific signatures for metabolites.

While for homogenous microbial cultures, protocols for rapid sample processing for immediate metabolome access are available, this needs still to be established for complex systems such as lichens. With current protocols at hand only stable metabolites such as secondary metabolites including those of bacteria (Boustie and Grube, 2005), polymeric compounds or compounds terminating metabolic build-up pathways may be reliably accessed and quantified.

One method to study metabolic pathways and fluxes of lichens *in vivo* is based on the utilization of stable isotope labeling. They are based on the supply of the labeled tracer to the growth medium (Kuhn et al., 2019). The positional labeling patterns can reveal the biosynthetic history of the studied products (Kinoshita et al., 2015). Labeling studies of intact lichens are challenging, because the symbiosis between photo- and mycobionts could trigger the metabolism of both (Calcott et al., 2018), but several projects engaged in this subject. Experimentation with ^14^C was popular in lichen physiology mainly for studying carbohydrate transfer (e.g., Richardson et al., 1968), but declined since then, while more recent studies favor the use of ^13^C in experiments. Lines et al. (1989) conducted ^13^C-NMR analysis and revealed the transport of ^13^C-photosynthate from the photo- into the mycobiont of *X. calciola* for mannitol biosynthesis. Sundberg et al. (1999) showed that there may be significant differences in carbon transfer rates and partitioning of carbon between the symbionts in different lichen (e.g., *L. pulmonaria*) by using ^13^C-NMR spectroscopy in combination with ^13^CO_2_ labeling. Aubert et al. (2007) identified 30 metabolites in *X. elegans* using ^13^C- and ^31^P-NMR spectroscopy and with that indicated that metabolite composition is affected by stress conditions. An additional study reveals that cyano- and tripartite lichens have a strong respiratory response to glucose by conducting carbon dioxide flux measurements and phospholipid acid analysis with experimental application of ^13^C_6_ (Campbell et al., 2013). Recently, Kuhn et al. (2019) performed *in vivo* labeling experiments with *Usnea dasypoga* using ^13^CO_2_ or [U-^13^CO_6_]-glucose and reconstructed the biosynthetic pathway of usnic acid.

Non-canonical amino acids (NCAAs), or “unnatural amino acids,” are identified in several microorganisms. Non-canonical D-amino acids (NCDAAs) are secreted by various bacteria as signaling molecules to aid the bacteria to cope with changing environmental conditions (Lam et al., 2009; Kolodkin-Gal et al., 2010; Leiman et al., 2013). Cava et al. (2011) indicate that NCDAAs cause biofilm dispersal in aging bacterial communities. NCAAs are additionally involved in the formation of cyanobacterial hepatotoxins. In recent studies, the hepatotoxins microcystin-LR and nodularin were frequently found in lichen cyanobionts (Oksanen et al., 2004; Kaasalainen et al., 2009, 2012). Such toxins can have grazing inhibition effects and therefore may protect the lichen against herbivory (Hrouzek, 2017). Labeling studies using NCAAs are known from several proteomic and biotechnological studies, because it benefits from the ability to enrich labeled proteins. Saleh et al. (2019) recently reviewed this method. However, to our knowledge this method was not used in lichen metabolite studies so far.

#### Molecular Imaging: Visualizing Interactions *in situ*

By combining the different omics approaches with imaging techniques previously overlooked participants, metabolites and the spatial occurrence of both can be found in lichens.

With fluorescence *in situ* hybridization (FISH) specific nucleic acid sequences can be detected and localized by using fluorescent probes. It can be used to uncover the taxonomical and spatial structure of bacterial communities in lichens (e.g., Cardinale et al., 2008; Muggia et al., 2013; Maier et al., 2014; Erlacher et al., 2015; Cernava et al., 2017). Levsky and Singer (2003) reviewed the methodology.

With matrix-assisted laser desorption/ionization (MALDI) imaging mass spectrometry (IMS), the distribution of proteins and small molecules within biological systems can be investigated by *in situ* analysis of thin tissue sections. It can determine the distribution of hundreds of compounds in only a single measurement and enables the acquisition of cellular expression profiles without destroying the cellular and molecular integrity. Cornett et al. (2007) and Walch et al. (2008) reviewed the methodology. In lichens, IMS can be used to visualize the distribution of secondary compounds, which was first shown by Le Pogam et al. (2016). Linking imaging mass spectrometry with metagenomics can determine the localization and organization of small molecules within the microbial community of lichens. For example, an ordered layering of molecules assigned to specific lichen symbiotic partners can be revealed (Garg et al., 2016).

### The Bacterial Community of *L. pulmonaria*

#### Where Bacteria Reside Within the Lichen Thallus

The surface of lichens, best studied in *L. pulmonaria* and *C. arbuscula*, is densely colonized by bacteria (Figure 1; Cardinale et al., 2008, 2012a, 2012b; Grube et al., 2009; Schneider et al., 2011). The density of bacteria on lichen thalli (e.g., *Cladonia rangifera*: c. 10^7^–10^8^ cells/g) is higher in relation to surfaces of higher plants (leaf surface: c. 10^5^ cells/cm^2^) (Cardinale et al., 2008; Grube et al., 2009; Saleem, 2015).

The Alpha diversities (Shannon index) of bacterial communities can vary between different lichens. *L. pulmonaria* shows an index of 7.0 whereas *Solorina crocea* an index of 4.5 (Grube et al., 2012; Aschenbrenner et al., 2014).

Lichen associated bacteria colonize distinct thallus parts in various abundances and patterns due to chemically and physiologically differences. Alphaproteobacteria are widespread on both the upper and lower surface of *L. pulmonaria* (Cardinale et al., 2012a; Grube et al., 2015). This is also demonstrated for other dorsoventrally organized lichen species, like *Umbilicaria* sp. (Grube et al., 2015). In contrast to Alphaproteobacteria, Betaproteobacteria are locally restricted on the lower surface (Grube et al., 2015). For *Cladonia* species, the highest amount of bacteria is found on the internal layer of the podetia (Cardinale et al., 2008, 2012b), whereas bacterial colonization on crustose lichens (e.g., *Lecanora* sp.) is higher in the cracks and fissures between the areoles of the crustose thalli (Grube et al., 2009), perhaps because humidity stays longer. Cardinale et al. (2008) found evidence for bacterial presence in cell walls of *C. arbuscula*, but not in the cytoplasm. Unsurprisingly, bacteria are also found on decaying lichen material of some terricolous lichens, like *Baeomyces rufus* or *C. rangiferina*, or in the lichen-substrate interface (Asta et al., 2001). In their study of the lichen genus *Xanthoparmelia*, Mushegian et al. (2011) found that the older central parts of this leaf-like lichen contain a richer and more stable bacteria community than the younger thallus periphery. This is partly confirmed by a study from Cardinale et al. (2012b) and Noh et al. (2020) for the upright thalli of *Cladonia* sp. The microbiome shows an increase in diversity from apical to basal parts and significant differences according to the vertical position within the thallus.

#### Microbiome Functions

##### Overview

**FIGURE 5:**
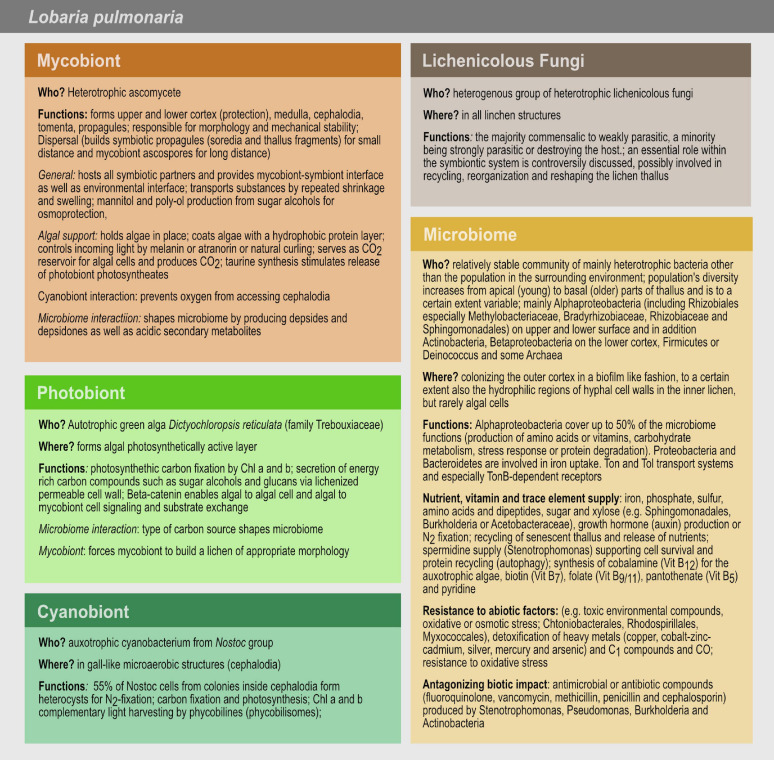
Microbiome and symbiotic partner’s functions in the lichen *L. pulmonaria*. The mycobiont is responsible for the holobionts’ morphology, mechanical stability and reproduction, protects and supports the photo- and cyanobiont and shapes the microbial community. The photobiont is in charge of the photosynthetic carbon fixation. The cyanobiont is responsible for nitrogen and carbon fixation. The detailed role of the lichenicolous fungi is unclear. The microbiome plays a role in nutrient, vitamin and trace element supply, in resistance to abiotic factors and in antagonizing biotic impact.

The lichen microbiome may contribute multiple functions to the lichen symbiotic system (Figure 5) (Hunter, 2006; Grube et al., 2015; Eymann et al., 2017). This includes essential functions such as nutrient supply by uptake and/or assimilation of e.g., iron, phosphate, sulfur, amino acids and dipeptides, sugar and xylose (e.g., Sphingomonadales, Burkholderia or Acetobacteraceae), resistance against abiotic factors (e.g., toxic environmental compounds, oxidative or osmotic stress; Chtoniobacterales, Rhodospirillales, Myxococcales), growth hormone production or nitrogen fixation (Figure 5; Liba et al., 2006; Grube et al., 2009, 2015; Schneider et al., 2011; Cardinale et al., 2012a; Erlacher et al., 2015; Cernava et al., 2017).

Grube et al. (2015) reveal that Alphaproteobacteria covered up to 50% of the microbiome functions in *L. pulmonaria*, e.g., production of amino acids or vitamins, carbohydrate metabolism, stress response or protein degradation. Proteobacteria and Bacteroidetes are involved in iron uptake. Ton and Tol transport systems and especially TonB-dependent receptors are revealed by metagenomic and –proteomic analyses. The amount of bacterial functions serving the self-preservation of the microbes or the entire lichen has to be elucidated in more detail in the future.

##### Uptake and supply of nutrients, essential compounds and trace elements

Bacteria can influence the growth of their host by e.g., producing growth hormones. This is supported by the identification of genes/proteins of the bacterial microbiome of *L. pulmonaria* (Grube et al., 2009) involved in auxin biosynthesis. The mobilization and recycling of material from senescent thallus parts may facilitate growth of the young and growing thallus parts (Grube and Berg, 2009; Grube et al., 2015; Cernava et al., 2017; Eymann et al., 2017).

*Granulicella* and *Lichenibacter* are lichen-associated bacteria for which carbohydrate use and production are known (Pankratov and Dedysh, 2010; Pankratov et al., 2019). The first one, the genus *Granulicella*, hydrolyzes pectin, xylan, laminarin and lichenan and produces an amorphous EPS matrix composed of polysaccharides in *Cladonia* lichens (Pankratov and Dedysh, 2010). *Lichenibacter* utilizes starch and xylan (Pankratov et al., 2019).

The role of bacterial strains (e.g., *Azospirillum*, *Bradyrhizobium* and *Frankia*) for supplements of fixed nitrogen to the symbiotic partners is obvious. Creating a partial anaerobic biofilm as bacterial habitat is important for bacterial nitrogen fixation, which may augment the nitrogen budget in lichens lacking a N-compound-donating cyanobiont. Several lichen-associated bacteria, like Alphaproteobacteria (Grube et al., 2009; Almendras et al., 2018), Gammaproteobacteria (Liba et al., 2006), Firmicutes (Grube et al., 2009; Almendras et al., 2018) and Actinobacteria (Almendras et al., 2018) contain *nifH* genes. In the case of N-limiting conditions, bacterial N-fixation, by e.g., *Azotobacter*, Betaproteobacteria or Alphaproteobacteria, could be of considerable importance for the vitality of lichens (Leveau and Preston, 2008; Grube and Berg, 2009; Grube et al., 2009; Bates et al., 2011; Erlacher et al., 2015). This fits well with studies of Almendras et al. (2018) showing that Chlorolichens have a higher diversity of N-fixing bacteria than cyanolichens. However, nitrogen supply is not a problem in many parts of Europe, as the atmosphere carries massive amounts of nitrogen compounds from agriculture over large distances.

Many algae are auxotrophic for vitamin B_12_ that is often synthesized by bacteria in symbiotic communities (Croft et al., 2005). Bacteria (e.g., Chtoniobacterales, Sphingomonadales, Sphingobacteriales, Myxococcales, and Rhizobiales) may be of importance for cofactor and vitamin synthesis and supplementation of the whole symbiotic system. Bacterial enzymes are found in *L. pulmonaria*, which are involved in the synthesis of cobalamin belonging to the Vitamin B12 group (relevant for photosynthesis), biotin (Vitamin B7; important for gene regulation), folate (Vitamin B9/B11; important for C_1_-metabolism), pantothenate (Vitamin B5; synthesis and degradation of carbohydrates) or pyridine (herbicide production) precursors supporting fungal growth (Erlacher et al., 2015; Grube et al., 2015).

We suggest that lichens, which can live up to hundreds of years, maintain a dynamic equilibrium of bacteria. The growing parts (alphaproteobacterial dominance) act as anabolic systems. The senescing parts might represent catabolic sinks. Cardinale et al. (2012b) assume that the bacteria, which are colonizing the older lichen parts, help to convert the old lichen biomass into simple molecules. These will then be released into the substrate or can be recycled to the growing lichen parts as it was shown previously (Ellis et al., 2005).

##### Abiotic stress and toxic compound protection

The bacterial microbiome of *L. pulmonaria* (e.g., Chthoniobacterales, Myxococcales, Sphingomonadales, Sphingobacteriales) shows resistance against abiotic stressors (Grube et al., 2015; Cernava et al., 2017), but the detailed mechanisms remain mostly unknown. Cernava et al. (2018) show that the microbial community structures do not depend on the level of arsenic concentration at the sampling site, whereas the functional spectrum related to arsenic metabolism is enhanced. Furthermore, the amount of detoxification related genes is higher in arsenic-polluted samples. Additionally, oxidative-stress protectants to heavy metal efflux are observed (Grube et al., 2015). Besides creating anaerobic niches, EPS and thus biofilm formation may play a role in the protection against pH and metals as shown for *Rhizobium leguminosarum*, a soil bacterium that establishes symbiosis with *Trifolium* spp. (Kopycińska et al., 2018).

Besides confirming the resistance against abiotic stressors, the bacterial microbiome may help with the detoxification of methanol/C_1_-metabolites. Formaldehyde-activating enzymes are highly abundant in bacterial samples, especially Methylobacteriaceae, of *L. pulmonaria*. These enzymes are involved in oxidation of methanol to carbon dioxide and formaldehyde detoxification. The assignment of carbon monoxide dehydrogenase to the same bacterial family in the metaproteomic analysis of *L. pulmonaria* indicates the existence of carbooxidotrophic bacteria or the detoxification of carbon monoxide, which competes with O_2_ for cytochromoxidase of the respiration chain (Eymann et al., 2017).

##### Antagonists of biotic impact

*Lobaria pulmonaria* and potentially other lichens may be important reservoirs for bacteria acting against bacterial and fungal pathogens. In former studies, it was shown that *Rhinocladiella* sp. and *Botrytis cinerea* were inhibited by bacterial strains isolated from *L. pulmonaria* (Cernava et al., 2015). *Rhinocladiella* are black fungi that may opportunistically act as human pathogens and could for example cause cerebral phaeohyphomycosis (neurotropic fungus *R. mackenziei*) by infecting nerve cells (Jabeen et al., 2011; Didehdar et al., 2015). *Botrytis cinerea is a plant pathogen* causing the gray mold disease (Williamson et al., 2007). The defense against such pathogens is possible by secretion of protective substances such as antimicrobial or antibiotic compounds (vancomycin, penicillin and cephalosporin). *Stenotrophomonas*, *Pseudomonas*, *Burkholderia* and Actinobacteria dominate the abundant antagonistic community of *L. pulmonaria* as producers of bioactive volatiles (Cernava et al., 2015, 2017).

#### Microbiome Acquisition and Shaping

**FIGURE 6:**
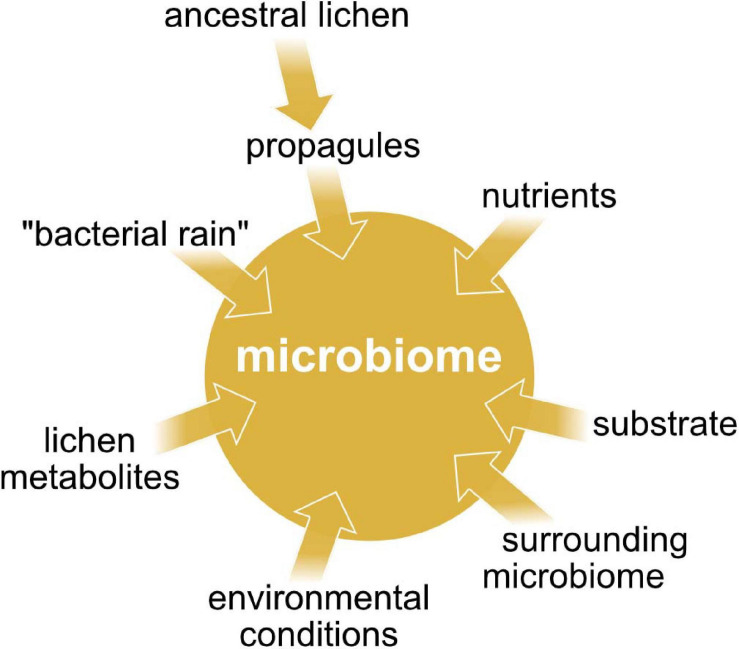
The main factors affecting the microbiome composition in lichens.

Little is known about the intraspecific variation of microbiome composition, and how lichens acquire their specific bacterial communities. A study of Aschenbrenner et al. (2014) indicate that the propagules of lichens contribute to a co-dispersal of lichen-associated bacteria (Figure 6), since the propagules (isidia) of *L. pulmonaria* share the overall bacterial community with the parental thalli at class level. This also suggests that the bacterial community structure might change over time at lower taxonomic ranks. Cardinale et al. (2012a) suppose that when propagules of *L. pulmonaria* are dispersed, the high-abundant *Alphaproteobacteria* are maintained for successful colonization of the new site. During colonization, both *Burkholderia* and nitrogen fixers will be lost, and local, better-adapted competitors may be picked up from the new environment.

The prokaryotic community may be recruited from “bacterial rain”, as for example known from phyllosphere microbiomes (Mechan Llontop, 2020), or from the adjacent substrate (Figure 6). Uptake of cyanobacterial photobionts from mosses growing around *L. pulmonaria* thalli was indicated by a study of Aschenbrenner et al. (2014). The mosses provide the cyanobacteria to the lichen propagules and young lichen thalli respectively. Cyanobacterial uptake from neighboring mosses was also demonstrated for the lichen *Erioderma pedicellatum* (Cornejo et al., 2005). Recently, Aschenbrenner et al. (2017) indicated that mosses growing around *L. pulmonaria* thalli may generally facilitate bacterial colonization of this lichen species. Additionally, they demonstrated that the bark microbiome of the inhabited tree species shares partly the same bacterial taxa with the lichen microbiome.

It is shown that the composition of the microbial communities is lichen specific (Grube et al., 2009; Bates et al., 2011; Hodkinson et al., 2012). On the lichens surfaces, a mixture of both abiotic and biotic conditions possibly controls bacterial growth. The poikilohydric conditions, with recurrent desiccation, may prevent the persistence of fast growing bacterial opportunists. Furthermore, it is known that rehydration following a desiccation period causes an oxidative burst by induction of high rates of superoxide production by the mycobiont. We argue this affects the bacterial microbiome or mycobiome and could select for survival of superoxide resistant bacteria, while the killed bacteria may serve as a nutrient source to support further lichen biomass production after rehydration.

Reports about antibacterial effects of lichen compounds or extracts (Burkholder et al., 1944; Ingólfsdóttir et al., 1998; Francolini et al., 2004; Boustie and Grube, 2005) suggest a biotic lichen originated control (Figure 6). These compounds may have antibiotic or rather antimicrobial properties and therefore may play an active role in selecting for specific types of bacteria. Among those biorelevant compounds you can find polyphenols such as depsides and depsidones, but also lactones, anthraquinones etc. (Hodkinson et al., 2012). Lichens producing substantial amounts of acidic secondary metabolites have significantly different bacterial communities than others (Grube and Berg, 2009). *L. pulmonaria* contains lower concentrations of such lichen-specific substances than many other Peltigerineae lichens (Beckett et al., 2003). Grube et al. (2015) hypothesize that bacterial colonization is mostly regulated by the mycobiont because they found only little evidence for quorum sensing in the microbiome of *L. pulmonaria*.

Besides secondary metabolites, the availability of nutrients may affect the microbial community (Figure 6). There is a significant correlation between photobiont-type and bacterial community composition. This is due to the major difference between cyanolichens and chlorolichens in the availability of fixed nitrogen (Hodkinson et al., 2012). Bacteria that are associated with cyanolichens have access to fixed nitrogen whereas bacteria of chlorolichens lack this benefit. Presumably, chlorolichens enrich bacterial species, which are capable of nitrogen fixation. Details of carbon release may play an additional role in shaping the prokaryotic community. Lichen-associated green algae release fixed carbon as sugar alcohols, contrary to glucose release of cyanobacteria. The bacterial utilization of sugar alcohols requires an adapted set of enzymes such as polyol dehydrogenases. For this reason, prokaryotic communities associated with chlorolichens consist of bacteria, which are able to synthesize sugar alcohol specific transporters and degrading enzymes while glucose degradation in cyanolichens does not need bacteria with adapted enzyme panels (Palmqvist, 2002; Adams et al., 2006; Hodkinson et al., 2012).

Metabolic processes can additionally modulate and organize complex microbial community structures by exchange of nutrients. Spatial concentration gradients of metabolites in biofilms lead to a varying nutrient availability and therefore could regulate the distribution of species (Cardinale et al., 2002; Stewart and Franklin, 2008). A diverse spectrum of chemical gradients can be established, like oxygen, nutrient or bacterial signaling compound gradients. The adaptation of the bacterial microorganisms to these gradients includes differences in gene expression and thus protein production (Stewart and Franklin, 2008). By competing for nutrients, metabolic tasks in a community can be divided. Therefore, the population can spatially differentiate in different sections such as metabolically active and inactive microbial cells (Liu et al., 2015). The exchange of nutrients is suggested to maintain genotypic diversity within naturally bacterial communities (Germerodt et al., 2016). To our best knowledge, there is no information about cross-feeding in lichen biofilms. As it is common in the microbial world (Pande et al., 2014), we suppose nutrient exchange also occurs in lichen biofilms.

#### Microbiome Diversity and Adaptability

Functional diversification in *L. pulmonaria*, due to a multiplayer network of the symbiotic partners supports longevity and persistence under changing environmental conditions (Grube et al., 2015). Lichen-associated bacteria seem to be influenced by climatically or geographically differences. The geographical patterning is defined by dispersal on a larger scale where host dispersal could be the limiting factor (Hodkinson et al., 2012). In *L. pulmonaria*, the diversity of Alphaproteobacteria is affected by geography. *Burkholderia* ssp. and nitrogen fixers are mostly taken up from the local environment (Cardinale et al., 2012a). Eymann et al. (2017) compared two different sampling sites of *L. pulmonaria* (Darß, Germany and Styria, Austria) and suggested the presence of a relatively stable core microbiome. There are differences in the distribution of families within the Alphaproteobacteria order Rhizobiales. Actinobacteria were more abundant in the samples collected from Darß and Acidobacteria and Planctomycetes were more abundant in the samples collected in Styria. The study indicates significant differences between the proteomes of the two lichen microbiomes in contrast to the rather stable fungal and algal protein profiles. For example, cold shock proteins were more abundant in the Styria lichen material. A study of Hodkinson et al. (2012) reveals that the major bacterial community is correlated with differences in large-scale geography. Printzen et al. (2012) found a less diverse microbial community of the lichen *Cetraria aculeata* in polar habitats. Antarctic and arctic communities are more similar to each other compared to samples from other regions such as Germany and Spain.

To understand how the extant microbiome responds to fluctuating environmental conditions in the natural habitats, Cernava et al. (2019) sampled lichens under representative hydration stages. Bacterial metatranscriptomes from *L. pulmonaria* reveal significant structural shifts and functional specialization corresponding to lichen hydration stages. The hydrated stage is correlated with upregulated transcription of transport systems, tRNA modification and various porins (Omp2b by Rhizobiales), whereas the desiccated samples suggest stress-adaptive responses. Carbohydrate metabolism is activated under both conditions, but under dry conditions, upregulation of a specialized ketone metabolism indicates a switch to lipid-based nutrition, reminiscent of ‘fasting metaorganism.’

The possibility that the bacterial microbiome composition and functionality change according to ecological and climatic variations, could lead to an increase in the adaptivity of the holobiont. The adaptation of lichen populations to new habitats is presumably accompanied by changes in the bacterial communities similar to previously observed switches of the photobiont strain correlating with different ecological conditions (Blaha et al., 2006; Grube and Berg, 2009). Photobiont switches can be beneficial for the mycobiont if the locally occurring photobiont strains are better adapted to the environmental conditions at a particular site than the carried photobiont strain. In such cases the adapted photobiont strain will be incorporated into the lichen during thallus establishment (Werth and Sork, 2010; Wornik and Grube, 2010; Werth and Scheidegger, 2012).

Klarenberg et al. (2020) provide evidence of compositional shifts in individual taxa of the microbiome of *Cetraria islandica* due to climatic warming. Warming alters the abundance of the most common taxa such as *Granulicella* or *Endobacter*. The abundance of *Granulicella* or *Bryocella* is decreasing whereas the abundance of *Acidisphaera*, *Sphingomonas* and *Endobacter* is increasing. After long warming periods bacterial microbiomes can acclimatize and therefore shift back to the original composition (Bradford et al., 2008; Crowther and Bradford, 2013; Romero-Olivares et al., 2017). Warming periods also affect the amount of lichen secondary metabolites and can therefore have an effect on the composition of the lichen microbiome. Usnic acid concentration is increasing during warming and perlatolic acid concentration is reduced (Asplund et al., 2017). Usnic acid acts as an antimicrobial protectant against fungal parasites, like *Fusarium moniliforme* (Cardarelli et al., 1997), which is involved in several human and animal diseases and produces different toxins (reviewed by Nelson, 1992). Despite that, usnic acid plays an additional role in protection against indicator bacteria such as *Staphylococcus aureus* or *Enterococcus faecalis* (Lauterwein et al., 1995). Perlatolic acid is also showing antibacterial, e.g., against *S. aureus* or *Escherichia coli* (Piovano et al., 2002; Gianini et al., 2008) and antifungal activities such as against *Cladosporium sphaerospermum* (Gianini et al., 2008).

Cardinale et al. (2012b) found out that lichens (e.g., *L. pulmonaria* or *C. arbuscula*) harbor higher numbers of bacteria when growing under shaded conditions. Despite that, lichens growing on rock harbor fewer bacteria than lichens growing on soil or bark. It seems that the amount of bacteria may be associated with the humidity of the habitat.

### Biotechnological Potential

Lichens tolerate extreme abiotic stressors (e.g., extreme climates or osmotic conditions) and accumulate toxic compounds, heavy metals or radionuclides. Therefore, they could be sources of biotechnologically interesting strains, compounds and enzymes (Davies et al., 2002, 2005; González et al., 2005; Grube and Berg, 2009). Most lichen secondary metabolites are of fungal origin, e.g., stictic acid as herbivory protectant (Elix and Stocker-Wörgötter, 1996), but evidence for a bacterial origin is already found. González et al. (2005) published a study focusing on lichen-associated Actinobacteria and their bioactivity. A large number of strains were obtained. Several strains belonged to the family Streptomycetaceae that is well known to produce bioactive compounds. Other actinobacterial families, which are also known for the production of bioactive compounds, such as Micromonosporaceae, Pseudonocardiaceae, and Thermomonosporaceae, were also isolated. About 30% of the strains showed antimicrobial activity against other microorganisms. The presence of some structurally identified bioactive molecules is reported for a few bacterial strains although there are many other strains of relevance (Cardinale et al., 2006; Liba et al., 2006; Grube et al., 2009; Selbmann et al., 2010; Pankratov, 2012; Kim et al., 2014; Lee et al., 2014; Sigurbjörnsdóttir et al., 2014; Cernava et al., 2015; Parrot et al., 2015). In the following paragraph, we summarize information about several lichen-associated bacteria producing already identified bioactive compounds.

An isolate of the dominant antagonistic genus *Stenotrophomonas*, found in the microbiome of *L. pulmonaria*, produces spermidine as the main bioactive compound (Cernava et al., 2015). Spermidine is a multifunctional polyamine that is a plant growth regulator, plays a critical role in plant embryo development and protects roots against stress (Al-Whaibi et al., 2012; Alavi et al., 2013). Additionally, spermidine affects biofilm formation in various bacterial species, like *Vibrio cholerae* (McGinnis et al., 2009), and acts as an antifungal biosynthesis regulator in *Lysobacter enzymogenes* (Chen et al., 2018). In eukaryotes, spermidine prolongs the life span and is known to play vital roles in cell survival, autophagy (the degradation of damaged and aggregated waste protein) and anti-aging (Eisenberg et al., 2009; Madeo et al., 2010, 2018). Tissue spermidine concentrations decline with age in model organisms (e.g., yeast, mice) as well as in humans (Scalabrino and Ferioli, 1984; Eisenberg et al., 2009; Pucciarelli et al., 2012; Gupta et al., 2013). Polyamines, like spermidine, can have procarcinogenic properties. Increased concentrations caused by enhanced biosynthesis can be found in different cancer types (Nowotarski et al., 2013). In contrast, cardiovascular diseases in humans and cancer manifestation in mice can be delayed by spermidine (Madeo et al., 2018). Dietary spermidine protects mice and probably also humans from cardiac aging, by e.g., improving the diastolic function or left ventricular elasticity (Soda et al., 2012; Eisenberg et al., 2016).

A *Streptomyces* strain, isolated from *C. uncialis*, produces the cytostatic enediyne uncialamycin, which shows strong antibacterial activity against the human pathogens *E. coli*, *B. cepacia* and *S. aureus* (Davies et al., 2005; Parrot et al., 2016). The same *Streptomyces* strain generates also the alkaloids Cladoniamides A-G that show toxicity against human breast cancer MCF-7 cells (Williams et al., 2008). Another *Streptomyces* strain produces the tetrapeptide lichostatinal that represents a cathepsin K inhibitor and is therefore of interest for the therapy of osteoporosis (Yasuda et al., 2005; Lavallée, 2011). Two other *Streptomyces* species producing novel cytotoxic compounds: chlorinated anthraquinonic angucycline (Motohashi et al., 2010) and aminocoumarines structurally closed to novobiocine (Cheenpracha et al., 2010) were isolated from lichen species. Furthermore, *S. cyaneofuscatus* synthesizes methacrylate derivates with cytotoxic effects (Parrot et al., 2016). Other isolates of this species also demonstrate with high potential to produce a variety of anthracycline family antitumor antibiotics daunorubicin, cosmomycin B, galtamycin B and the antifungal macrolactam maltophilin (Ando et al., 1985; Jakobi et al., 1996; Antal et al., 2005). *Actinoplanes* sp. ATCC55532 produces actinoplanic acids A and B. The latter inhibits farnesyl protein transferase and the farnesylation of the oncogene protein Ras. Thus, it has a potential for the treatment of colorectal carcinoma, exocrine pancreatic carcinoma and myeloid leukemia (Singh et al., 1997). Beside the discovery of novel bioactive lead compounds, direct antagonistic effects of bacteria of the lichen microbiome can be used as biological plant protection by defending plants against fungal plant pathogens, e.g., *Alternaria alternata* or *Phytophthora infestans* (Grube et al., 2009, 2012; Gasser et al., 2011; Kim et al., 2013a, 2014).

Despite the production of small metabolites, lichen-associated bacteria also show other biotechnological potentials. Many of these bacteria have the potential to produce PHA biopolymers and show high antagonistic potential against plant pathogens, like *A. alternata* (Gasser et al., 2011; Kim et al., 2013b, 2014). This is also mentioned in section “Microbiome Acquisition and Shaping.”

## Concluding Remarks

The morphological structure of the lichen thallus may affect the organization of the symbiotic networking. Metaorganisms such as lichens consist of highly integrated partnerships reflecting the classical dual definitions of the lichen symbiosis, and less tightly integrated partners with auxiliary functions (associated microbiome) (Hyvärinen et al., 2002; Cornejo and Scheidegger, 2013). In order to fully understand the microbiome contribution to the lichen symbiosis from a metabolic perspective, metabolomics could inform about the modes of nutrient exchange between the participating organisms. A question not yet properly addressed is, for example, whether bacteria thriving on the polysaccharides of the lichen mycobionts actively secrete certain compounds, or whether material from degrading bacteria is passively reabsorbed by the mycobiont. Degrading of bacteria might be forced by oxidative bursts, a well-known phenomenon from lichens in response to rehydration following desiccation (Minibayeva and Beckett, 2001). Selective bacterial degradation through oxidative bursts recalls not only a similar way of nutrient acquisition described from the plant rhizosphere (Paungfoo-Lonhienne et al., 2010), but could also exert selective pressure on the bacterial lichen colonizers, favoring species, which cope particularly well with oxidative stress. However, experiments like carbon isotope labeling and comparative physiological analysis between lichens with and without the bacterial microbiota are difficult to assess due to slow metabolism and high bacterial diversity. Additionally, variation in the structure of the lichen holobiont or histochemical variation of lichen extracellular matrix due to bacterial colonization should be incorporated in the analyses of the lichen symbiotic model. Correlations between the compositions of the bacterial microbiome, climatic changes and fungal/algal genotypes would reveal new insights into the functionality of the bacterial microbiome, its acquisition and influence on the lichen holobiont.

Certainly, more work is required to understand the complex interplay between lichens and their bacterial colonizers.

## Author Contributions

All authors contributed to the writing of the manuscript. MGru, JB, and US created the figures.

## Conflict of Interest

The authors declare that the research was conducted in the absence of any commercial or financial relationships that could be construed as a potential conflict of interest.

## Footnotes

1 www.lichenicolous.net
